# Cryo-EM tomography and automatic segmentation delineate modular structures in the postsynaptic density

**DOI:** 10.3389/fnsyn.2023.1123564

**Published:** 2023-04-06

**Authors:** Jae Hoon Jung, Xiaobing Chen, Thomas S. Reese

**Affiliations:** Laboratory of Neurobiology, National Institute of Neurological Disorders and Stroke, National Institutes of Health, Bethesda, MD, United States

**Keywords:** cryo-EM tomography, postsynaptic density, automatic segmentation, modular organization, nanodomain

## Abstract

Postsynaptic densities (PSDs) are large protein complexes associated with the postsynaptic membrane of excitatory synapses important for synaptic function including plasticity. Conventional electron microscopy (EM) typically depicts PSDs as compact disk-like structures of hundreds of nanometers in size. Biochemically isolated PSDs were also similar in dimension revealing a predominance of proteins with the ability to polymerize into an extensive scaffold; several EM studies noted their irregular contours with often small granular structures (<30 nm) and holes. Super-resolution light microscopy studies observed clusters of PSD elements and their activity-induced lateral movement. Furthermore, our recent EM study on PSD fractions after sonication observed PSD fragments (40–90 nm in size) separate from intact PSDs; however, such structures within PSDs remained unidentified. Here we examined isolated PSDs by cryo-EM tomography with our new approach of automatic segmentation that enables delineation of substructures and their quantitative analysis. The delineated substructures broadly varied in size, falling behind 30 nm or exceeding 100 nm and showed that a considerable portion of the substructures (>38%) in isolated PSDs was in the same size range as those fragments. Furthermore, substructures spanning the entire thickness of the PSD were found, large enough to contain both membrane-associated and cytoplasmic proteins of the PSD; interestingly, they were similar to nanodomains in frequency. The structures detected here appear to constitute the isolated PSD as modules of various compositions, and this modular nature may facilitate remodeling of the PSD for proper synaptic function and plasticity.

## Introduction

The postsynaptic density (PSD) was first observed by electron microscopy (EM), as an electron-dense structure lining the postsynaptic membrane opposed to the presynaptic active zone in asymmetric synapses (Palay and Palade, 1955; Palay, 1956; Gray, 1959; Whittaker and Gray, 1962; Akert et al., 1969). This large protein complex, typically observed at the tip of dendritic spines, has since been the subject of intense studies by EM and other techniques, and it is widely known that the PSD contains neurotransmitter receptors and critical elements of postsynaptic plasticity and is linked to the actin cytoskeleton and adhesion proteins in the synaptic cleft (see reviews Boeckers, 2006; Sheng and Kim, 2011; Harris and Weinberg, 2012; Dosemeci et al., 2016).

Conventional EM studies of plastic embedded PSDs in thin sections have described PSDs as disk-like structures with diameters typically within about 200–1,000 nm range and thickness less than 100 nm *in vivo* (Palay and Palade, 1955; Gray, 1959; Cohen and Siekevitz, 1978). PSD fractions biochemically isolated from brain have been used to identify key proteins of the PSD such as PSD-95; they were also reported to show morphologically similar structures (Cotman and Taylor, 1972; Cotman et al., 1974; Blomberg et al., 1977; Cohen et al., 1977; Carlin et al., 1980).

During the last decade, advances in super-resolution microscopy allowed imaging of several PSD components in live cells. The new imaging techniques revealed clusters of receptors and other PSD components, called nanodomains or nanoclusters, which appear to be dynamic and travel around within the PSD region (MacGillavry et al., 2013; Nair et al., 2013; Broadhead et al., 2016; Hosokawa et al., 2021). These studies pointed out the existence of discrete complexes within the confines of the PSD. Interestingly, several EM studies of isolated PSDs noted the presence of granular or globular structures ranging from 5 to 30 nm within the PSDs, using visual assessment (Carlin et al., 1980; Petersen et al., 2003; Swulius et al., 2012); however, structures greater than those structures in size, comparable to a majority of those nanodomains within isolated PSDs, remained unidentified. In a recent study, we reported further fragmentation of isolated PSDs upon mechanical disruption by ultrasound (Dosemeci et al., 2019); conventional EM images of the separated PSD fractions showed structures of 40–90 nm in size disconnected from intact PSDs, called PSD fragments, apparently derived from complexes of PSD proteins. The observation of PSD fragments led to a proposition that the PSD is organized as a patchwork of complexes loosely associated to each susceptible to mechanical disruption. The proposition and those nanodomains reported by super-resolution microscopy studies raise the possibility that isolated PSDs contain discernable substructural components similar to PSD fragments and nanodomains in size including those granular or globular structures.

To test the possibility, here we employed cryo-electron tomography (cryo-EM tomography) on isolated PSDs; we also developed and applied an automatic segmentation approach for visualization and delineation of PSDs and their substructures. Cryo-EM tomography was used to produce tomographic reconstructions of fully hydrated PSDs close to their physiological condition without artifacts due to embedding and fixation-induced cross-linking that could blur or obscure separation of substructures at a few nanometer resolutions in 3D (Dubochet and Sartori Blanc, 2001; Baumeister, 2002; Al-Amoudi et al., 2004). While manual segmentation has been successfully used for deciphering particular synaptic ultrastructures (Chen et al., 2008; Cole et al., 2016; Jung et al., 2021), structural analysis of isolated PSDs has been limited to visual assessment and manual measurement; here we implemented a new strategy of automatic segmentation for efficient and objective delineation of the entire PSDs and their substructures. As a preliminary step toward the delineation of the whole PSD in 3D, we developed an automatic segmentation optimization method (ASOM), expediting segmentation of the PSD by automatically denoising the segmented PSD. For automatic delineation of their substructures, we employed a 3D watershed segmentation method. Watershed segmentation has been used in various segmentation tasks to separate touching or connected structures such as segmentation of cells and is well-suited for the segmentation of molecular machines containing proteins and other macromolecules (Beucher, 1979; Volkmann, 2002; Lucic et al., 2016).

Our automatic segmentation delineated electron-dense structures within isolated PSDs, revealing individual substructures, also called modules here, and showing that a large portion of the modules regardless of sonication are similar in size to PSD fragments, which were produced upon sonication and seen by conventional EM in our previous study (Dosemeci et al., 2019); we also found smaller structures less than 40 nm in size similar to those from other EM studies (Carlin et al., 1980; Petersen et al., 2003; Swulius et al., 2012) and those greater than 90 nm. Furthermore, we found that several modules, called “*trans*-PSD modules” here, span the entire thickness of the PSD, presumably representing protein complexes linking membrane associated proteins such as PSD-95 to cytoplasmic proteins like actin proteins and that they are similar to those nanodomains in frequency. Our findings support the modular organization of the PSD and also suggest that modular structural components or modules within the PSD are of weak binding and various size, which may contribute to remodeling of the PSD important for synaptic function and plasticity.

## Materials and methods

### Preparation of PSD fraction from rat brain

Brains from 7 to 12 weeks-old rats of both genders were custom collected and immediately frozen in liquid nitrogen by Rockland (Gilbertsville, PA). PSD fraction from cerebral cortex was prepared as described previously (Dosemeci et al., 2019). Briefly, the synaptosome fraction was treated with 0.5% Triton X-100. The detergent-insoluble pellet was separated and further fractionated by sucrose density centrifugation and a crude PSD fraction was collected from the 1.5 M/2.1 M sucrose interface. After a second extraction with 0.5% Triton X-100 in 75 mM KCl, the PSD fraction was collected over a 2.1 M sucrose cushion.

### Sonication

A probe sonicator, Kontes KT50 micro ultrasonic cell disruptor (frequency 20 KHz), was used for sonication. The PSD preparation was resuspended in 20 mM HEPES pH 7.4 to a final concentration of 0.5 mg protein/ml. Reaggregation of mechanically separated components (750 μl) was sonicated four times at an output amplitude setting of 40%, each 20 s sonication followed by a brief cooling interval on ice.

### Preparation of samples for cryo-EM tomography

Vitrification of freshly prepared PSD fraction was performed on Vitrobot (Mark IV, Thermo Fisher Scientific, Waltham, MA, USA) to fix the PSD fraction in its hydrated state for direct visualization of the PSD and its substructures using cryo-EM tomography. A 3 μl drop of the PSD fraction was placed onto the freshly glow discharged 200 mesh Quantifoil copper grid and with 3 μl of 10 nm colloidal gold with a blotting time 3–4 s, and then plunged into liquid ethane (Dubochet et al., 1988; Fernandez-Busnadiego et al., 2011). Vitrified samples were stored in liquid nitrogen before imaging.

### Electron tomography

#### Data collection

Cryo-EM tomography data were collected with single-tilt using a Titan Krios (FEI) with a K2 for sonicated PSDs and K3 Summit direct electron detector (Gatan) for unsonicated PSDs. The Titan Krios was operated at an acceleration voltage of 300 kV and Gatan image filter (GIF). Images were collected in counting mode. Tilt series were acquired from −60 to +60° at 2° intervals using the data acquisition software SerialEM (Mastronarde, 2005), with the defocus value maintained at −4 μm, and the total accumulated dose of 60–80 e^–^/Å^2^. The pixel size was 0.3514 nm, and the image size was 3,938 × 3,710 pixels for sonicated PSDs while for unsonicated PSDs, the pixel size was 0.327 nm and the image size was 5,760 × 4,092 pixels. Despite the slight difference in pixel size between the sonicated and unsonicated PSDs, all the images were taken to completely include those PSDs to minimize any impact that may result from the difference.

### Reconstruction

Tilt series were aligned, downsampled by a factor of two, and reconstructed using IMOD (Kremer et al., 1996), ImageJ (Schindelin et al., 2012), or an upgraded version of EM3D^1^ (Ress et al., 1999; Harlow et al., 2001; Jung et al., 2021). Gold beads added to the sample before plunge freezing were used as fiducial markers to align the tilt series. For tilt series containing more than two fiducial markers, images were aligned by the markers and reconstructed by a simultaneous iterative reconstruction technique (SIRT) (Frank, 2006) using IMOD, generating sonicated datasets. For tilt series containing insufficient markers, images were aligned by cross-correlation using ImageJ and reconstructed by a weighted backprojection using EM3D, generating unsonicated datasets, because use of ImageJ and EM3D for alignment and reconstruction was simple and generated reconstructed volumes providing the quality visually comparable to the nearly optimal alignment and reconstruction by IMOD. Note that reconstructed volumes of PSDs showing disk-like structures and typical PSD sizes ranging from 200 nm to 800 nm were selected (two sonicated and six control PSDs) for the following segmentation, surface model generation, and analyses.

### Segmentation and rendering surface models

The selected PSDs were manually segmented throughout reconstructed volumes using EM3D because EM3D provides a simple tool to draw a region containing a structure of interest with only a few anchor points on each of the slices that includes the structure of interest throughout the reconstructed volume. Because the PSDs were highly electron-dense and irregular, their volumes of interest (VOIs) were defined by manually marking a closed path on the series of slices where their PSDs was completely enclosed. Typically, the VOIs were slightly larger than the structures of interest to allow optimal surface models of the structures. The manual delineation of VOIs generating nearly optimal surface models of the structures within the VOIs is tedious and time-consuming. Here instead of such challenging manual segmentation and refinement of VOIs for generating their nearly optimal surface models, we combined the manual segmentation method with the automatic segmentation optimization method (ASOM), which we developed, and a 3D watershed segmentation method for automatic segmentation of PSD substructures because watershed segmentation has been used widely applied to a variety of biological image segmentation tasks separating connected objects (Volkmann, 2002; Cheng and Rajapakse, 2009; Burette et al., 2012; Atta-Fosu et al., 2016; Lucic et al., 2016; Kiss et al., 2017).

### Automatic segmentation

We developed ASOM, automatically reducing noise in segmented PSDs, thus, significantly reducing the time and efforts for segmentation of PSDs in tomograms; we also used 3D watershed method for automatic segmentation of substructures in the PSD (see Supplementary Appendix).

### Measurements of PSDs and modules

In order to measure the size and area of each of the PSDs, the centroid and three orthogonal, principal axes of each of the segmented PSDs along the three eigenvectors were computed using the covariance matrix of coordinates forming the surface of the PSD similar to previous publications (Jung et al., 2016, 2018, 2021). The lengths of the PSD along the three axes were measured, and the shortest length among the three distances was determined to be the thickness of the PSD here. The size of each of the modules was determined by measuring the longest length among the three distances for the module. The area of the PSD was determined to be the extent of the projected area along the direction of the axis giving the shortest distance of the PSD. The volume of each of the PSDs was determined by counting the number of voxels in the PSD and multiplying the number to the cubed pixel size of the reconstructed volume containing the PSD. The volume of each of the modules was determined in the same way. *Trans*-PSD modules that span the whole thickness of the PSD were determined by finding modules directly connected to both external surfaces of the PSD across the thickness of the PSD.

### Statistical analyses

Kolmogorov–Smirnov (KS) tests, one-way ANOVA tests, and Spearman rank correlations were performed with OriginPro software package (OriginLab, Northampton, MA, USA). Significance was defined as *p* < 0.01. All the averages were given with their standard deviation (SD) and presented as means ± SD.

### Computer hardware and software

For the analyses, a PC computer was used loaded with Windows 10, Interactive Data Language (IDL) software package (Exelis, Boulder, CO, USA), EM3D coded in IDL, and EM3D, coded in C++ and Java, that was recently upgraded by Jae Hoon Jung (NINDS) and Eun Jin Choi (NINDS), were used. IDL and C++ were used to implement ASOM and the 3D watershed segmentation method.

## Results

**FIGURE 1:**
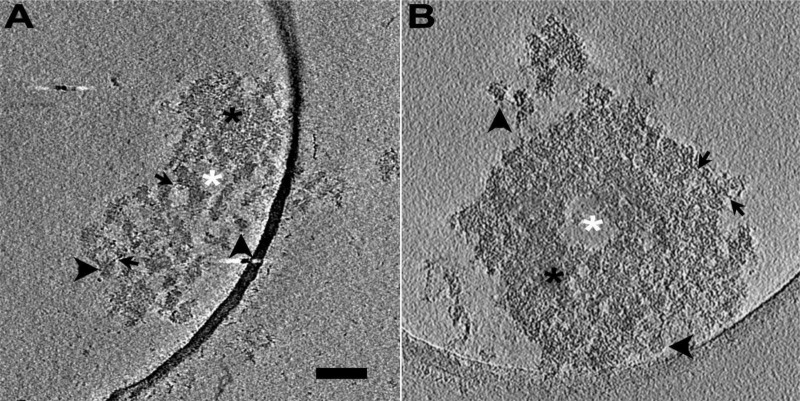
Isolated PSDs with and without sonication, imaged by cryo-EM tomography. **(A)** A 1.4-nm-thick virtual slice through a reconstructed volume of a sonicated PSD. The slice shows electron-dense structures apparently completely isolated from other structures (vertical black arrowhead), those apparently incompletely disconnected from other structures showing a few connections with adjacent ones though they were noticeably away from each other in most of their parts (horizontal black arrowhead), other apparently condense structures (black asterisk), and empty spaces devoid of such structures (white asterisk) including filamentous structures (black arrow). **(B)** A 1.3-nm-thick virtual slice through a reconstructed volume of an isolated PSD without sonication (control PSD). The slice also shows electron-dense structures apparently completely isolated from other structures (horizontal black arrowhead), those incompletely isolated from other structures (vertical black arrowhead), other apparently condense structures (black asterisk), and empty spaces devoid of such structures (white asterisk) including filamentous structures (black arrow). Scale bar = 100 nm.

Here we observed intact PSDs from PSD fractions biochemically isolated from rat brain (control PSDs) and those followed by application of sonication (sonicated PSDs) by cryo-EM tomography (Figure 1). On individual images of the sonicated PSDs, some globular structures seemed completely isolated without any noticeable connection to others (see structures pointed by vertical arrowheads in Figure 1A); for some other structures, most of their parts were isolated similar to those completely isolated ones, but these structures showed a few connections with others (see structures pointed by horizontal arrowheads and black arrows in Figure 1A); the condensed structures showed a size of multiples of those globular structures; filamentous structures were also often observed (see structures pointed by arrows in Figure 1A). Control PSDs showed all the structures and empty space similarly (Figure 1B) while empty spaces were more frequently observable from sonicated PSDs.

**FIGURE 2:**
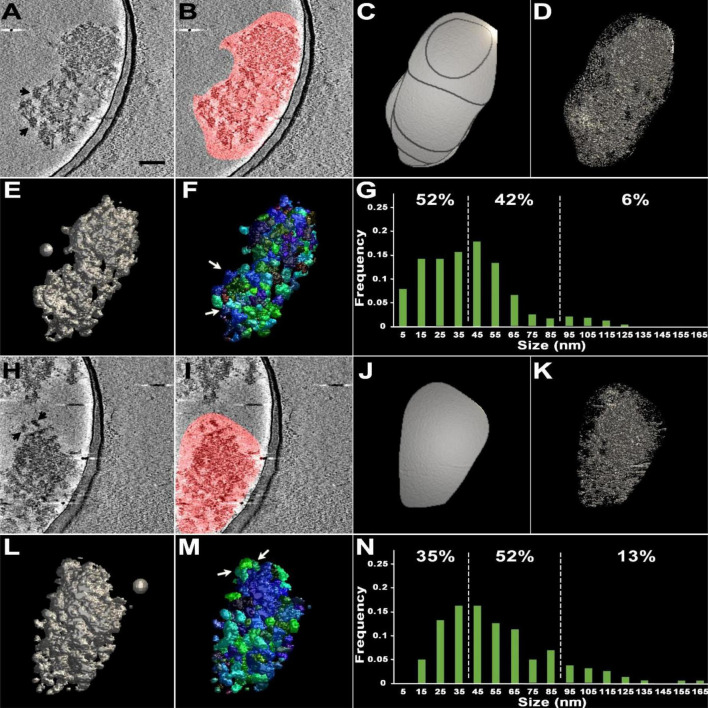
Cryo-EM tomography and subsequent segmentation of two sonicated PSDs. **(A,H)** A virtual slice (2.8 nm thick) of each of two different PSDs through their reconstructed volumes. Black arrows point to clearly delineated structures within the PSDs likely to be loosened upon sonication. Scale bar = 100 nm. **(B,I)** Virtual slices with regions of interest containing PSDs marked in red on the two-dimensional slices, segmented by hand without any repetitive manual refinement. **(C,J)** A surface model of the volume of interest (VOI) containing each of the PSDs by manual segmentation. Here, outlines of segmented regions on slices form lateral surface of the VOI; the outlines of the first and last slices form the top and bottom bases of the VOI, showing that modification of the VOI is required to obtain the properly segmented PSD. **(D,K)** A surface model of each of the segmented PSDs after lowering the isodensity level to reduce noise. Note that the models are still highly noise-embedded. **(E,L)** A surface model after application of ASOM onto each of the VOIs markedly reducing the noisy structures automatically. Signal-to-noise ratios increased from 0.93 to 1.9 for panel **(D)** and from 0.10 to 0.51 for panel **(K)**. **(F,M)** Surface models of differently color-coded substructures or modules within each of the PSDs, obtained by 3D watershed segmentation. White arrows point to the automatically segmented modular structures corresponding to those visually noted in panels **(A,H)** by black arrows. **(G,N)** Histograms showing size distribution of modules within the PSDs. White dotted lines indicate the range from 40 to 90 nm. The spheres of 50 nm in diameter in panels **(E,L)** were included as a guidance of the scale.

Distinct electron-dense complexes similar to fragments were occasionally detected upon visual inspection of tomographic slices of these PSDs (Figures 2A, H). With the aim of visualizing individual substructures in the PSDs, the segmentation of two chosen PSDs was carried out first by manually outlining the PSD on each of the slices in the reconstructed volume that contains the PSD in a rudimentary way, creating their volumes of interest (VOIs) (Figures 2B, C, I, J). Surface models of the hand-segmented PSDs exhibited a high noise level (Figures 2D, K). To remove the noise, ASOM, our method supporting effective segmentation, was applied to the PSDs (Figures 2E, L). In the resulting renderings, the surfaces of the PSDs appeared highly irregular, rough, and lumpy, reflecting a patchy morphology and supporting that the PSDs are composed of highly interconnected substructures along with some loosely connected ones of various sizes.

To delineate the substructural feature more readily and objectively, we used a 3D watershed method, suited for separating connected or adjacent 3D structures, which was validated by comparing easily notable substructures segmented manually with those segmented by the 3D watershed method (Figures 2A, F, H, M and Supplementary Figure 1). The application of the watershed method generated hundreds of separate substructures within the PSDs (Figures 2F, M). We determined the size of each of the substructures by measuring their longest length (Figures 2G, N; see also Section “Materials and methods”). The majority of segmented substructures in sonicated PSDs (>92%) were within a range of 10–170 nm in size, and we call these structures *modules* (Figures 2G, N). The broad size range includes the presence of small modules (<30 nm) similar to those granular or globular structures in size, which were noted within isolated PSDs in several EM studies (Carlin et al., 1980; Petersen et al., 2003; Swulius et al., 2012). We also found that more than 40% of those modules fell within the 40–90 nm range of previously reported PSD fragments (Dosemeci et al., 2019). The average size of the modules of the sonicated PSDs was about 50 nm (53.4 nm ± 3.9 nm, *n* = 384).

**FIGURE 3:**
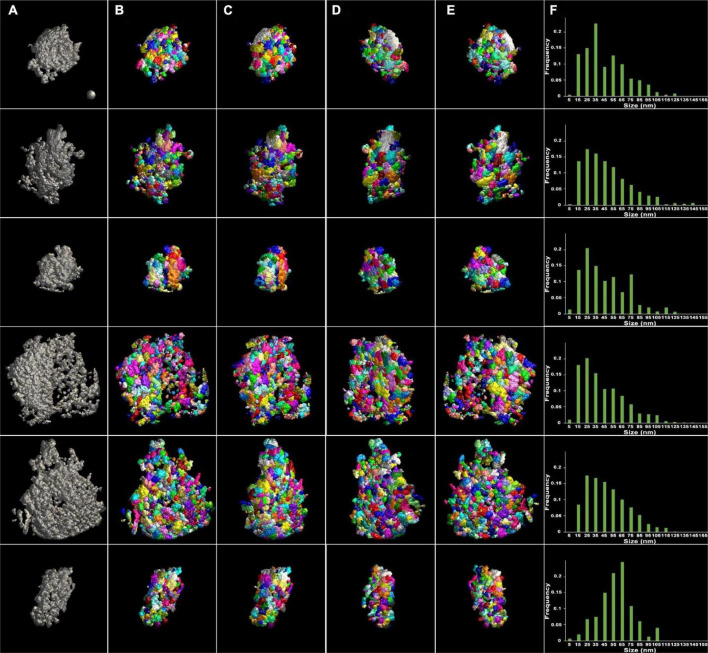
Surface models of unsonicated PSDs and their modules viewed at four different angles after segmentation. **(A)** Surface models of six different PSDs in gray after application of ASOM, visualized *en face*. The surfaces of the PSDs were irregular and lumpy. Holes or gaps were present in some of the PSDs (see the fourth and fifth PSDs). The PSDs separated into modules by the 3D watershed segmentation and color-coded were viewed **(B)** *en face*, **(C)** after ∼45-degree rotation, **(D)** after ∼135-degree rotation, and **(E)** from behind. **(F)** Histograms of the size of PSD modules. The distributions were asymmetric and skewed to the left except for one distribution (sixth distribution). Most modules were less than 125 nm in size, and the average size was ∼50 nm. A sphere of 50 nm in diameter in the first panel **(A)** was included as a guidance of the scale.

The similarity of the size to that of PSD fragments in the previous study suggests presence of discrete, module-like complexes, within the PSDs, that become partially detached from one another upon mechanical disruption. Alternatively, segmented modules could result from mechanical disruption breaking PSDs at random fissures in the structure. To test this possibility, control PSDs were analyzed in the same way. In their reconstructed volumes, the control PSDs appeared to contain patches of material differing in electron density delineating modular structures (see Figure 1). As before, surface models of the PSDs were constructed using ASOM, and segmentation of modules was carried out by the watershed method. In control samples, six PSDs were chosen for analysis. These PSDs were typical in morphology and size, ranging from 200 nm to 800 nm in diameter (Figure 3; Tables 1, 2). There was no significant difference between the volumes of the PSDs and the sonicated PSDs (8.6 × 10^6^ nm^3^ ± 5.2 × 10^6^ nm^3^ for the control PSDs; 7.0 × 10^6^ nm^3^ ± 3.9 × 10^5^ nm^3^ for the sonicated PSDs; *p* = 0.86, KS test) and also no difference between their areas (1.8 × 10^5^ nm^2^ ± 1.1 × 10^5^ nm^2^ for the control PSDs; 1.3 × 10^5^ nm^2^ ± 3.1 × 10^4^ nm^2^ for the sonicated PSDs; *p* = 0.86, KS test). The results indicate that PSDs similar to control PSDs are also present after the sonication. Control PSDs were variable in size; however, the sizes of modules fell in a common range and showed a similar distribution, with the exception of one PSD shown in Figure 3F (*p* = 0.012, one-way ANOVA for the first five distributions; *p* < 0.001, one-way ANOVA for all the six distributions). Then the PSDs were watershed-segmented to detect their modules. Although all the size distributions of the modules for their corresponding PSD were not the same, most modules (>97%) were within a size range of 10–170 nm similar to those of the sonicated PSDs. The broad size range also includes the presence of small modules (<30 nm) similar to those granular or globular structures in size (Carlin et al., 1980; Petersen et al., 2003; Swulius et al., 2012). Furthermore, more than 38% of them fell within the size range of PSD fragments (40–90 nm) similar to those of the sonicated PSDs. The average size of the modules for the control PSDs was about 50 nm (48.3 nm ± 4.9 nm, *n* = 2,058). The size distribution of the control PSDs was not statistically different from that of the sonicated PSDs (*p* = 0.21, KS test) indicating that the sonication does not generate any significant change in the size distribution of the modules.

**TABLE 1:** Areas, thicknesses, and volumes of control and sonicated PSDs.

PSDs	Area (10,000 nm^2^)	Thickness (nm)	Volume (10,000 nm^3^)
**Control**			
PSD1	10.71	154.6	1,205
PSD2	16.17	161.4	807.5
PSD3	8.125	99.29	408.3
PSD4	28.85	110.3	600.7
PSD5	33.23	141.7	1,737
PSD6	8.836	192.4	428.3
**Sonicated**			
PSD1	14.99	124.0	730.5
PSD2	10.64	142.5	675.9

**TABLE 2:** Volumes and sizes of *Trans*-PSD modules from control and sonicated PSDs.

PSDs	Volume (10,000 nm^3^)	Size (nm)
**Control**		
**PSD1**		
*Trans*-PSD Module 1	33.27	132.31
*Trans*-PSD Module 2	28.42	141.48
*Trans*-PSD Module 3	18.16	114.232
*Trans*-PSD Module 4	11.64	107.944
*Trans*-PSD Module 5	16.76	109.516
*Trans*-PSD Module 6	8.831	103.49
*Trans*-PSD Module 7	6.007	105.586
*Trans*-PSD Module 8	7.733	103.752
**PSD2**		
*Trans*-PSD Module 1	52.34	106.634
**PSD3**		
*Trans*-PSD Module 1	26.44	128.118
*Trans*-PSD Module 2	9.694	93.272
*Trans*-PSD Module 3	14.44	114.232
*Trans*-PSD Module 4	10.97	117.638
*Trans*-PSD Module 5	14.12	101.918
**PSD4**		
*Trans*-PSD Module 1	18.34	120.52
*Trans*-PSD Module 2	19.24	127.594
**Sonicated**		
**PSD1**		
*Trans*-PSD Module 1	12.38	121.24
*Trans*-PSD Module 2	10.14	105
**PSD2**		
*Trans*-PSD Module 1	46.32	168.84
*Trans*-PSD Module 2	43.25	139.44
*Trans*-PSD Module 3	28.98	153.44

The computational method used here allowed analysis of the volumes of whole PSDs (the sonicated and control PSDs) as well as modules. The volumes of individual PSDs under analysis were measured by counting the number of voxels of each of the PSDs after applying ASOM as described in Methods and are shown in Table 1. Volumes of modules varied within a range of 4.7 × 10^2^–5.2 × 10^5^ nm^3^. Although small modules are commonly identified from all the PSDs, relatively large modules (≥40 nm) make up 89% or more of the volume. Similar to the size distributions of modules of the control PSDs, the distributions of their volumes were not statistically different with the exception of one PSD (*p* = 0.10, one-way ANOVA only for the first five distributions; *p* < 0.001, one-way ANOVA for all the six distributions). We also found that there is a significant correlation between the number of the modules and the volume of their PSD (*p* < 0.001, Spearman rank correlation; see Supplementary Figure 2). The number of the modules also correlated with the area of their PSD (*p* < 0.001, Spearman rank correlation; see Supplementary Figure 3). These correlations show that the modules scale in number with respect to their PSD area and volume.

**FIGURE 4:**
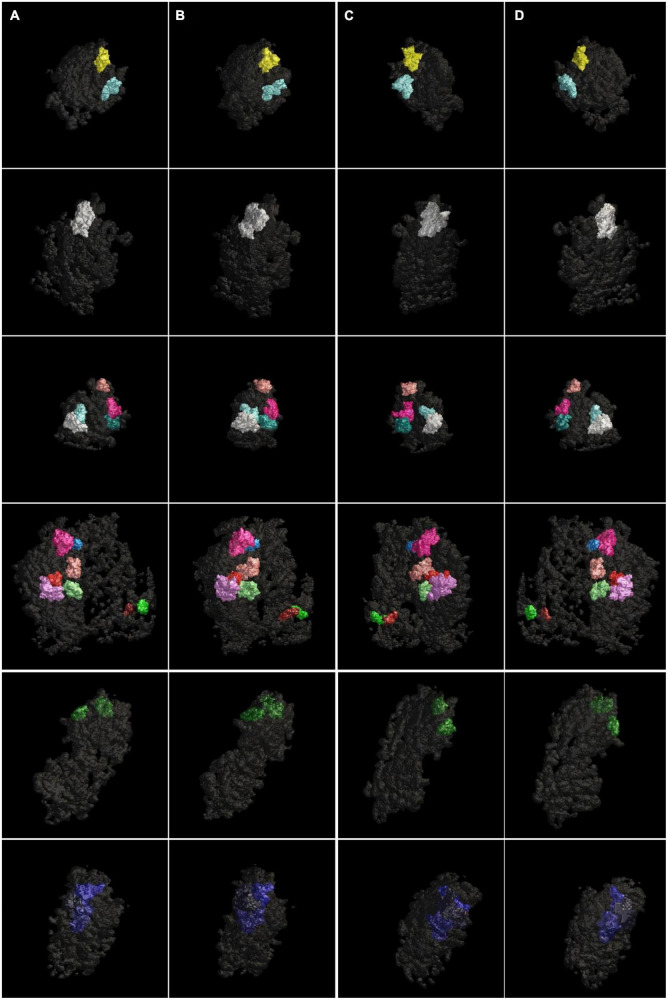
Surface models of modules spanning the whole thickness of the PSDs (*Trans*-PSD modules) viewed at four different angles. The *Trans*-PSD modules of four different PSDs were viewed **(A)** *en face*, **(B)** after ∼45-degree rotation, **(C)** ∼135-degree rotation, and **(D)** from behind. The PSDs contained one to eight *Trans*-PSD modules. Each *Trans*-PSD module fell into a range from 6 × 10^4^ nm^3^ to 5 × 10^5^ nm^3^ in volume taking up 0.5–6% of the volume of its PSD for control PSDs (four rows from the top) and from 1 × 10^5^ nm^3^ to 5 × 10^5^ nm^3^ in volume taking up 1.4–7% for sonicated PSDs (two rows from the bottom).

Our watershed segmentation detected modules of electron-dense material within the PSD supporting existence of discrete multi-protein complexes. We were especially interested in those modules that span the whole thickness of the PSD, as they are likely to link proteins in the synaptic cleft to the actin cytoskeleton in the cytoplasm. Indeed, such large modules, called *Trans*-PSD modules here, were detected from four out of six control PSDs (*n* = 16) and from the two sonicated PSDs (*n* = 5) as shown in Figure 4 and Table 1. Of PSDs containing *Trans*-PSD modules, the number of *Trans*-PSD modules from the control PSDs ranged from one to eight (Figure 4; Table 1) and two to three in sonicated PSDs (Figure 4; Table 1). The number distribution of *Trans*-PSD modules was not statistically different between control and sonicated PSDs (*p* = 0.86, KS test) indicating that the number of *Trans*-PSD modules is not affected by the sonication. The volumes of individual *Trans*-PSD modules were in the range of 6.0 × 10^4^–5.2 × 10^5^ nm^3^ (0.5–6% of the PSD volume) for control PSDs and 1.0 × 10^5^–4.6 × 10^5^ nm^3^ (1.4–7% of the PSD volume) for sonicated PSDs (Table 2). The volume distributions of the *Trans*-PSD modules of control and sonicated PSDs were not statistically different (*p* = 0.28, KS test) indicating that the volumes of the *Trans*-PSD modules are unaffected by the sonication. The sizes of the *Trans*-PSD modules ranged from 93 nm to 141 nm for control PSDs and from 105 nm to 169 nm for sonicated PSDs (Table 1); the size distributions of the *Trans*-PSD modules between control and sonicated PSDs were not statistically different (*p* = 0.15, KS test). The percent of the total volumes of the *Trans*-PSD modules ranged from 6.3 to 19% for the control PSDs and 3.1–18% for sonicated PSDs indicating that the volume of the PSD can be taken by *Trans*-PSD modules up to ∼20%. The results show that the *Trans*-PSD module varies in number, size, and volume, but distributions of their number, size, and volume remain the same after the sonication suggesting that the *Trans*-PSD module is composed of protein complexes unaffected by the sonication.

## Discussion

Here cryo-EM tomography and our automatic methods (ASOM and 3D watershed segmentation) were used to examine the substructure of isolated PSDs. We found the PSDs comprise modules mostly tens of nanometers in size, regardless of sonication for mechanical disruption of weakly bound modules; furthermore, we found modules spanning across PSDs; from those followed by application of sonication, we also found similar results indicating that those modular structures are stable enough to be unaltered in size by the sonication.

The cryo-EM technique allows observation of proteins in their hydrated form. A few studies on cultured hippocampal synapses from embryonic rats imaged by Cryo-EM tomography visualized PSDs of tens of nanometers thick present on the cytoplasmic side of the postsynaptic membrane and observed that PSDs contain short filaments protruding from the postsynaptic membrane (Lucic et al., 2007; Tao et al., 2018). The observation is consistent with those PSDs and filaments observed from hippocampal cultures from embryonic rats prepared by high-pressure freezing, freeze substitution, heavy metal staining and resin infiltration and imaged by electron tomography (Chen et al., 2008; Cole et al., 2016). As observed by the cryo-EM technique, isolated PSDs in this study exhibit disk-like shapes with irregular contours, similar to those reported by other EM techniques but appear thicker (> ∼100 nm) as noted previously (Swulius et al., 2012; Farley et al., 2015); filaments connected to the postsynaptic membrane were not observable from the isolated PSDs because the membrane was not preserved, but filamentous structures were often observed within the isolated PSDs (Figure 1). In the absence of chemical fixation and heavy metal staining, areas of differing density were apparent, indicative of modules of the PSD in our tomographic slices (Figure 1), consistent with our previous study (Dosemeci et al., 2019). Observation of these discrete subcomplexes in cryo-EM tomograms gave us the impetus for segmentation of the structures.

A key step for the successful segmentation of PSDs and their substructures has been the development of an automatic segmentation of the structures. Synaptic structures of neuronal cultures and synaptosomes in mammalian central nervous system have been segmented by hand (Chen et al., 2008; Fernandez-Busnadiego et al., 2011; High et al., 2015; Cole et al., 2016; Tao et al., 2018; Jung et al., 2021) while some of those structures such as molecular complexes in the synaptic cleft or membrane bound complexes were reported to be segmented by automated procedures (Lucic et al., 2005, 2016). Several methods and procedures have been developed or applied to segment prominent cellular/subcellular structures or pleomorphic membrane-bound molecular complexes (Volkmann, 2002; Cyrklaff et al., 2005; Lucic et al., 2005, 2016; Lebbink et al., 2007; Sandberg and Brega, 2007; Mishchenko, 2009; Moussavi et al., 2010; Rigort et al., 2012; Weber et al., 2012; Martinez-Sanchez et al., 2014; Perez et al., 2014; Page et al., 2015; Chen et al., 2017); 3D watershed segmentation was reported to be effective in identifying membrane structures from a reconstructed volume containing the Golgi region in a pancreatic beta cell after reducing noise and also to be possible to segment actin filaments and also actin monomers (Volkmann, 2002). Despite the advancement of automatic segmentation, substructural features within the isolated PSDs imaged by cryo-EM tomography have been limited to visual assessment. Our automatic approach using ASOM and 3D watershed segmentation allowed automatic segmentation of the PSDs and their substructures for their quantitative analyses that have not be available so far.

The loosening of the PSD structure in sonicated samples is interpreted as simple mechanical dissociation of weak protein-protein interactions by ultrasound (Dosemeci et al., 2019). Automatic segmentation of these sonicated PSDs as well as control PSDs by ASOM and watershed in this study yielded a patchwork of modules (Figures 2, 3) of ∼50 nm on average. Interestingly, the average size of a module within the PSDs analyzed here is similar to our previously reported average size of the PSD fragments, fragments distant from sonicated PSDs (Dosemeci et al., 2019), and this similarity supports that the PSD fragments, if not all, are the modules detached from a PSD. Considering the estimated mass density of the PSD, ∼10 kDa/nm^2^ (Chen et al., 2005), the mass of a module based on its average size (50 nm) is expected to be approximately ∼20 MDa. Furthermore, if we assume that the average mass of a protein is ∼100 kDa, then the average-sized module is expected to be possible to contain more than 100 proteins, showing that a majority of the modules are large enough to contain complexes of multiple proteins such as those constituting the core layer of the PSD lining the postsynaptic membrane and others in an additional layer or pallium (Dosemeci et al., 2019). A few EM studies noted granular or globular structures less than 30 nm within isolated PSDs (Petersen et al., 2003; Swulius et al., 2012). Consistent with the studies, we found small modules similar to those structures and also those greater than the PSD fragments in size (see Figures 2, 3). Furthermore, we found *Trans*-PSD modules that span the whole thickness of the PSD and that the number of *Trans*-PSD modules is small compared to that of other modules (see Figures 3, 4). We also found that PSDs contain various numbers of these *Trans*-PSD modules (Table 1). *Trans*-PSD modules are relatively large, ranging from 0.5 to 7% of their PSD volume. Considering 1.1 GDa as a total mass of an average PSD (Chen et al., 2005), *Trans*-PSD modules are expected to be at least ∼50 MDa, and this shows that they are likely to contain complexes of many proteins more than average-sized modules. Furthermore, their span of the entire PSD thickness suggests that they may contain elements from both the PSD core and pallium elements, with PSD-95 proteins anchoring glutamate receptors into scaffolding supported by Shank and Homer scaffold and linked to the pallium *via* guanylate kinase-associated proteins (GKAPs) and other linker proteins (review: Dosemeci et al., 2016) while other modules may contain only the PSD core or pallium elements. Thus, *Trans*-PSD modules may represent important functional units joining membrane components to the actin cytoskeleton while other modules may represent disjoined PSD elements playing different functional roles. It is probable that conventional fixation and staining protocols inhomogeneously stain discrete complexes and/or obscure their separation within the PSD, and, indeed, to our knowledge, such entities have not been observed in EM studies using these techniques although structural components of the PSD core such as filaments, ∼20 nm long, vertically extending from the postsynaptic membrane and other filaments, ∼20 nm or ∼35 nm long, oriented parallel to the postsynaptic membrane were revealed by our previous EM tomography study (Chen et al., 2008). A study by high pressure freezing without aldehyde fixative (Rostaing et al., 2006) reported filamentous projections extending to the spine cytoplasm. Another study (Burette et al., 2012), however, using an osmium-free protocol showed pyramidal structures with a base of ∼60 nm within the PSD, with apexes toward the spine cytoplasm contacting the actin cytoskeleton. These structures coming from the PSD and going deep into the cytoplasm raise a possibility that there is a structure crossing from the side of the synaptic cleft of the PSD to the side of the cytoplasm of the PSD. Indeed, we found *Trans*-PSD modules that are large enough to reach both surfaces of the PSD suggesting that filamentous projections and pyramidal structures may have a close relationship with the *Trans*-PSD modules described here.

Several super-resolution microscopy studies showed clustered PSD proteins and their dynamic lateral movements (Fukata et al., 2013; Nair et al., 2013; Tang et al., 2016; Hruska et al., 2018). Such clusters and movement might be accounted for by the modular organization of the PSD and weak interactions between modules as suggested here. The numbers of nanodomains, called also nanoclusters or nanomodules, measured in the studies were not the same with each other. A few super-resolution microscopy studies reported one to four nanodomains or nanomodules of PSD-95 (Fukata et al., 2013; Hruska et al., 2018) and about two nanodomains of PSD-95 (Tang et al., 2016) while zero to six nanodomains of AMPA receptors were reported (Nair et al., 2013), showing that the number of nanodomains is expected to be between zero and six based on the studies. Interestingly, the number of *Trans*-PSD modules measured in this study is within the range of those measured by the super-resolution microscopy studies. Measured sizes of nanodomains from the studies broadly varied; however, several studies reported that average sizes of nanodomains of a few key proteins of the PSD are close to ∼100 nm (MacGillavry et al., 2013; Nair et al., 2013), which is also similar to the sizes of our *Trans*-PSD modules (see Table 2). The similarities in the number and size suggest that the *Trans*-PSD modules found in this study may be closely related to the clusters of PSD proteins although this unexpected similarity is required to be investigated further in future to determine their direct inter-relationship. Furthermore, the size of immunogold clusters labeling AMPA receptors in rat hippocampal neurons was reported to range from 30 nm to 160 nm by an immunogold EM study (Nair et al., 2013); the range of the size is also similar to that of the segmented modules in this study (Figures 2, 3) suggesting that not only *Trans*-PSD modules but also other modules may contain key PSD proteins or protein complexes although *Trans*-PSD modules and other modules may exhibit dissimilar degrees of mobility contributing to the maintenance and alteration of the PSD organization differently.

Our findings support a dynamic modular organization of the PSD where modules are in various sizes containing complexes of PSD elements; modules can stay stably in place, move around, be removed, or added for playing an important role on regulating synaptic transmission and plasticity, consistent with other studies (MacGillavry et al., 2011; Dosemeci et al., 2019). The commonality of modules for all the PSDs examined here and the finding of *Trans*-PSD modules, similar to those nanodomains in number and size, and other modules similar to those small granular or globular structures noted by several studies suggest that our modular organization of the PSD may have general applicability in understanding synaptic organization and function of synapses.

## Data availability statement

The raw data supporting the conclusions of this article will be made available by the authors, without undue reservation.

## Author contributions

JJ and TR designed the research and wrote the manuscript. JJ and XC performed the research. JJ analyzed the data. All authors contributed to the article and approved the submitted version.

## Footnotes

1
em3d.org
