# Supplementary material for: Cryo-EM tomography and automatic segmentation delineate modular structures in the postsynaptic density
Source: Front Synaptic Neurosci. 2023 Apr 6;15:1123564. doi: 10.3389/fnsyn.2023.1123564 (PMC10117989; doi:10.3389/fnsyn.2023.1123564)
Supplement: Supplementary file 1 [file Data_Sheet_1.pdf]

Appendix

Automatic Segmentation Optimization Method (ASOM).

Cryo-EM tomograms are composed of grayscale images typically with a high level of noise (Bepler et al., 2020). In those images of the tomograms, noise tends to show a random fluctuation in the grayscale intensity without showing any noticeable structures of interest. If our structures of interest are prominent such as membranes or membrane enclosed structures like synaptic vesicles and mitochondria, filtering can be effective reducing the level of noise. However, if our structures of interest are not as prominent as those, there is a possibility that filtering can distort structural details in the images regarding those structures of interest. Furthermore, our isolated PSDs and their modules are likely to be different from each other, preventing us from using any subtomogram averaging technique to obtain their high-resolution structures (Wan and Briggs, 2016). Hand segmentation of isolated PSDs and their modules are also challenging because the surfaces of isolated PSDs and their modules are highly irregular. Several automatic segmentation methods and procedures have been developed (Chen et al., 2017, Cyrklaff et al., 2005, Lebbink et al., 2007, Lucic et al., 2016, Lucic et al., 2005, Martinez-Sanchez et al., 2014, Mishchenko, 2009, Moussavi et al., 2010, Page et al., 2015, Perez et al., 2014, Rigort et al., 2012, Sandberg and Brega, 2007, Volkmann, 2002, Weber et al., 2012); however, they were designed for prominent cellular/subcellular structures or pleomorphic membrane-bound molecular complexes. To effectively segment isolated PSDs, we have developed Automatic Segmentation Optimization Method (ASOM) that enhances the signal to noise ratio (SNR) in our VOIs by improving a method previously developed for automatic segmentation of synaptic structures of high contrast (Jung and Szule, 2017), combining thresholding and a variable-weight 3D box filter to make it applicable to cryo-EM tomograms as described in the following.

A VOI of a structure of interest was created by manually delineating the boundary of a structure of interest with multiple anchor points that enclose a structure of interest on a series of virtual slices through a reconstructed volume using EM3D. Similarly, a VOI of the background was created by putting multiple anchor points around a region that has no distinguishable structure on a series of images.

The signal-to-noise ratio (SNR) of the VOI was defined as

$$\text{SNR} = \frac{|\mu_O - \mu_\text{B}|}{\sigma_\text{B}} \quad (1)$$

where μ_O is the mean grayscale value of the VOI of a structure of interest, and μ_B and σ_B are the mean and standard deviation of the grayscale values of a background VOI, respectively. Note that as SNR increases, the noise in the VOI decreases.

We applied thresholding using a value between the minimum and mean grayscale values of the background VOI. The value was typically the sum of the minimum greyscale value and 70% of the difference between the minimum and mean grayscale values because with the value, the boundary of the segmented structure appeared nearly optimally located, and it was adjusted, if necessary, depending on the noise level of each of them in the tomograms. Thus, any voxel greater than the threshold grayscale value was considered as noise and any other voxel as signal. However, we found that application of this thresholding onto a structure VOI tended to generate a highly noisy structural surface model that contained tiny structures of a few voxels in size (see Fig. 2D and 2K). To remove those tiny noisy structures automatically, we developed a novel 3D box filtering technique. After thresholding, the structure VOI was scanned by a cubic box of a few voxels in size. During the scan, the ratio of the number of signal voxels to all the voxels in the box was computed. If the ratio is equal to or less than a threshold ratio, ranging from the average ratio of the background VOI to one, then all voxels in the box were considered as noise; otherwise, they were considered as signal. This reduces noise voxels within the structure VOI. In this way, a new automatically optimized VOI was generated by enhanced SNR, containing only voxels classified as signals. With this process, signal can be enhanced by a factor of two or more (see Fig. 2D-E and 2K-L) depending on the values of the parameters adjusted by a user. Note that ASOM was applied to all the hand-segmented PSDs to obtain the PSDs presented and analyzed here.

3D watershed segmentation.

Watershed segmentation is one of most common segmentation methods used in processing biological and material science images (Atta-Fosu et al., 2016, Beucher, 1979, Burette et al., 2012, Cheng and Rajapakse, 2009, Furat et al., 2019, Kiss et al., 2017, Lucic et al., 2016, Neumann et al., 2019, Volkmann, 2002). In watershed segmentation, an image is considered as a landscape having ridges and valleys, of which the elevation values are typically defined by the grayscale values of the pixels in the image. And filtering has been typically applied before watershed transformation to mitigate incorrect segmentation that can be generated by incomplete

or inhomogeneous boundary of structures of interest. Reconstructed volumes obtained by cryo-EM tomography have been widely known to have a low SNR at high resolutions (Sali et al., 2003). Thus, we first downsampled reconstructed volumes by a factor of two to the power of a whole number to enhance the SNR, prior to the application of watershed, making the dimension of the voxel of all the reconstructed volumes about 2.5 × 2.5 × 2.5 nm. Because we are interested in applying watershed segmentation onto our segmented PSD in 3D not the entire tomogram, the VOI of each of the PSDs optimized by ASOM was distance-transformed replacing the grayscale value for each voxel with the nearest distance to the background to allow separation of connected structural components of the PSD. Next, local maxima of the distances were computed for the distance-transformed PSD. Then, these local maxima were used as seed points. The distance-transformed VOI was 3D watershed-transformed, expanding the seeds in size. This produced separate objects of individual structural components of the PSD. The lower volume limit (3×3×3 voxels ≅ 25 voxels) was imposed here to discard small structures that were present dominantly in the background. We found that watershed-segmented structures similar to visually notable and manually segmented modules in the PSD are also observed (see Figs. 2A, 2F, 2H, 2M, and S1) indicating that our automatic approach effectively segments visually notable modules of the PSD. Note that when there was no downsampling used or downsampling generated the dimension of the voxel smaller than about 2.5 × 2.5 × 2.5 nm, watershed-segmented structures situated at or near the positions of those manually segmented modules were notably smaller than those manually segmented ones. There were no other procedures than the downsampling and ASOM taken, prior to our watershed segmentation for automatic segmentation of modules in the PSD.

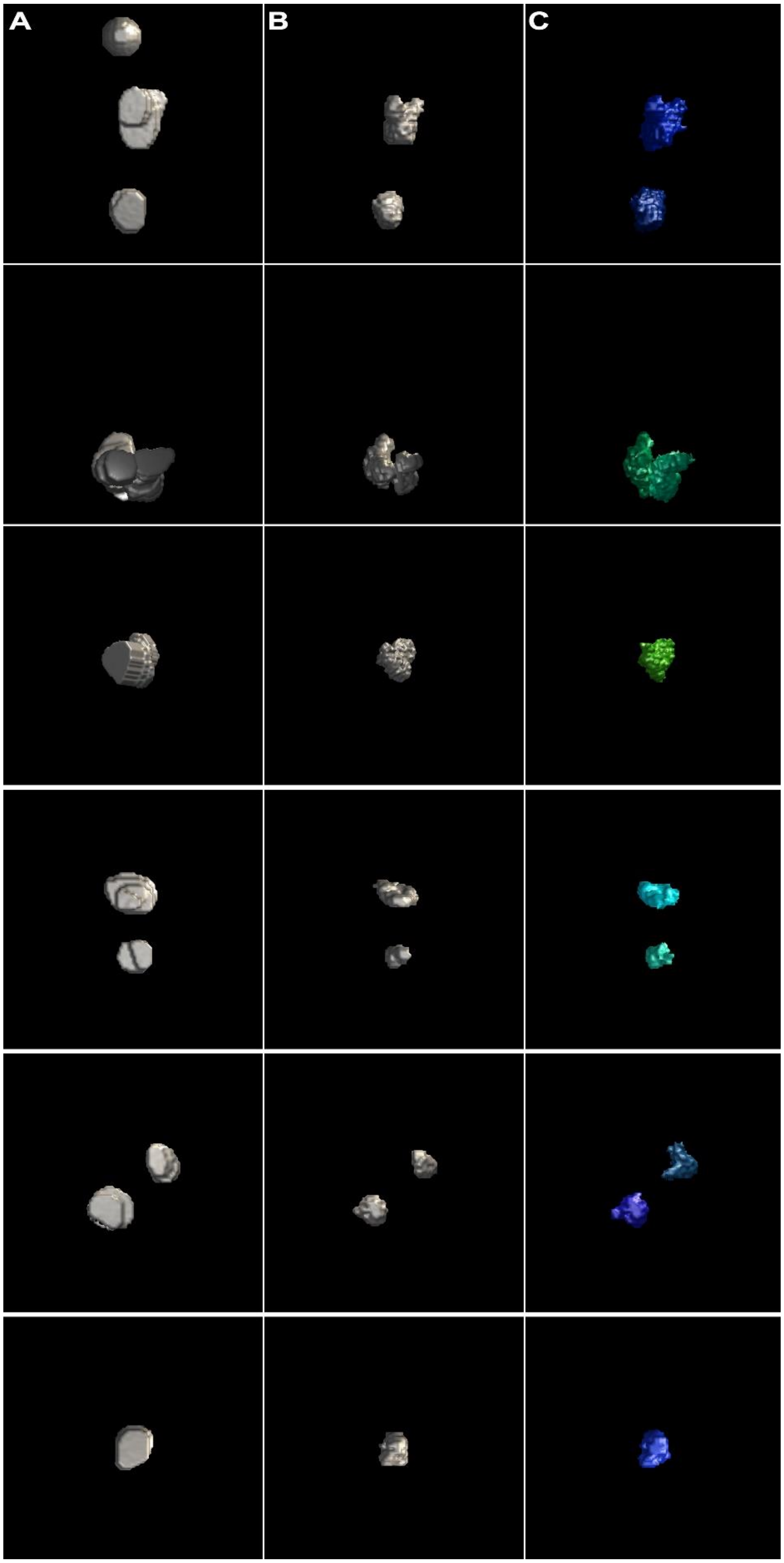
A
B
C

S1. Comparison of manual segmentation of modules contained in PSDs and the 3D watershed segmentation of structures corresponding to them. (A) Surface models of two modules, indicated by the black arrows in Fig. 2A, those indicated in Fig. 2H, and other modules generated after segmentation of them by manually marking a closed path on the series of slices where each of the module was completely enclosed similar to manual segmentation of the PSDs as described in Methods. (B) Surface models of the structures generated after application of ASOM. (C) Surface models of structures, generated by the 3D watershed segmentation, that are located at the same locations with those in A. The watershed segmented structures are similar to the structures in B although watershed segmentation was applied onto their ASOM-applied PSD. Furthermore, it should be noted that the watershed segmentation of hundreds of modules within a PSD took only a few hours demonstrating the efficiency of our 3D watershed segmentation combined with ASOM. A sphere of 50 nm in diameter in A was included as a guidance of the scale.

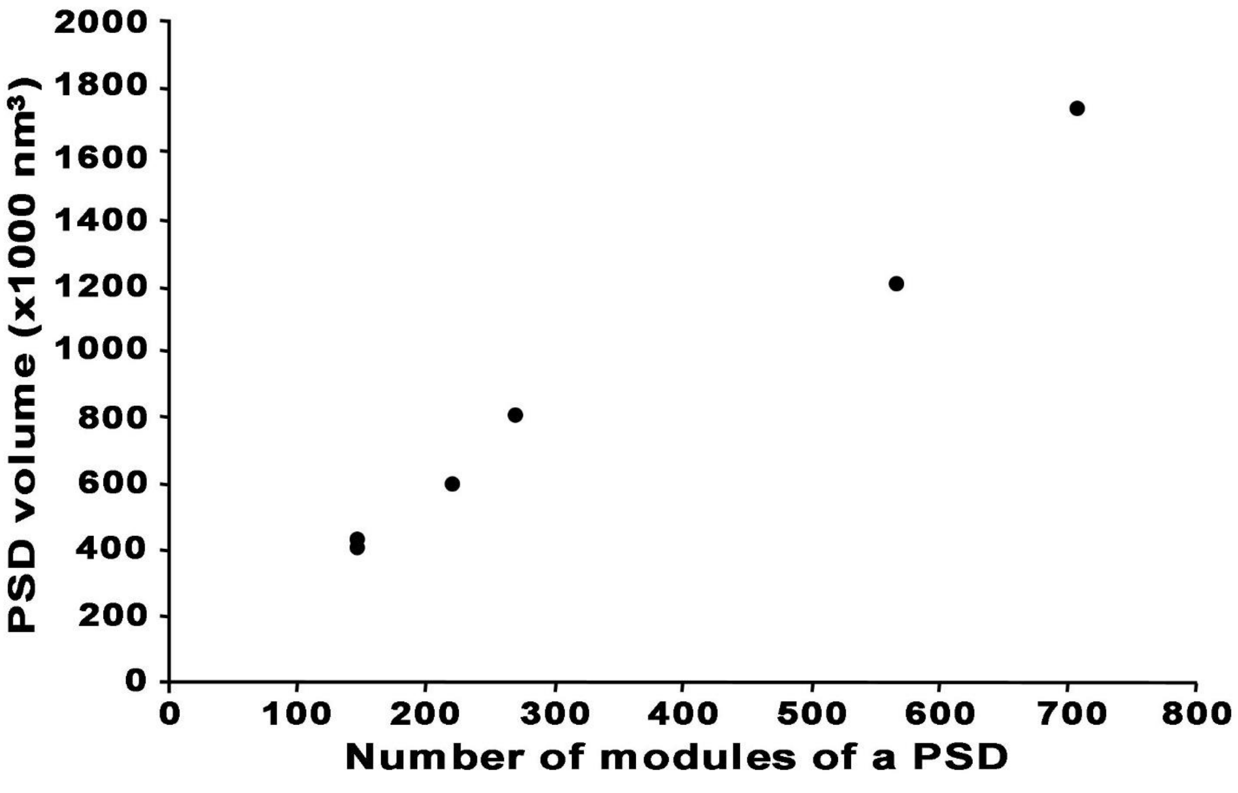

S2. Correlation between the PSD volume and the number of modules of a PSD.
The volume of a PSD shows a positive correlation with the number of modules of the PSD for the six control PSDs ($p < 0.001$, Spearman rank correlation).

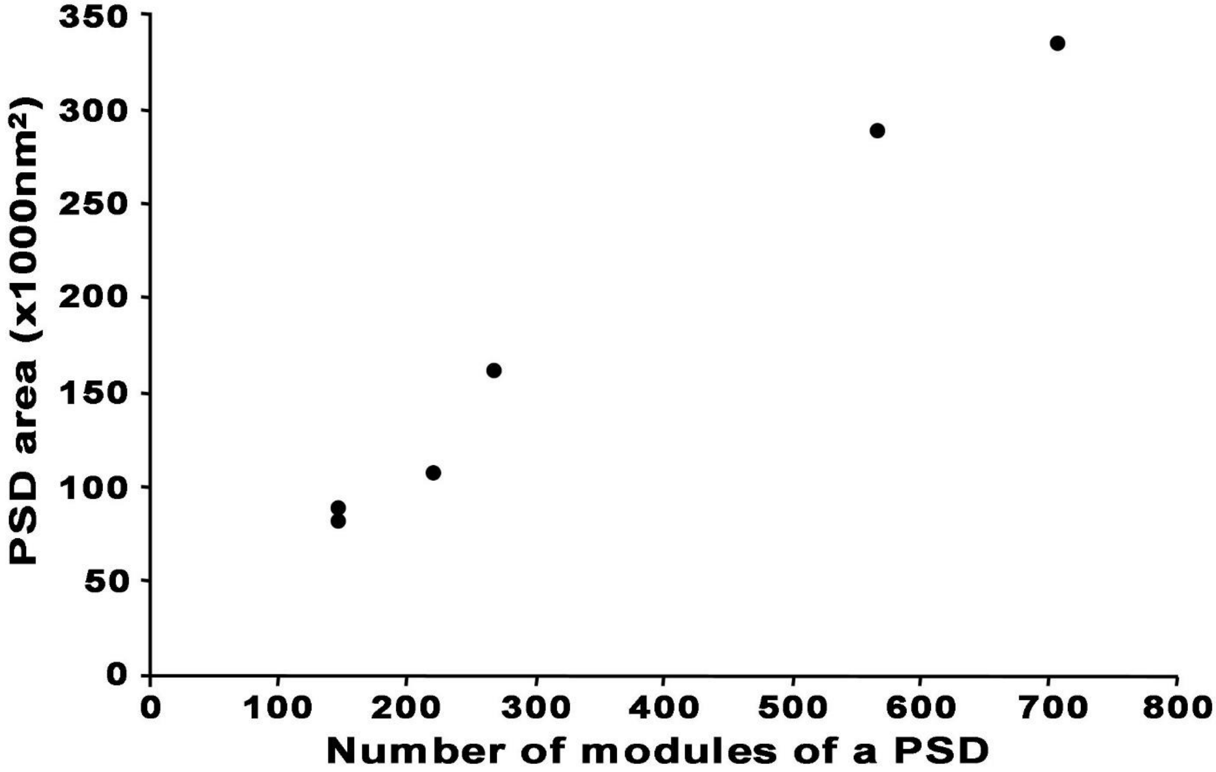

S3. Correlation between the PSD area and the number of modules of a PSD.

The area of a PSD shows a positive correlation with the number of modules of the PSD for the six control PSDs ($p < 0.001$, Spearman rank correlation).
